# Ex-vivo recellularisation and stem cell differentiation of a decellularised rat dental pulp matrix

**DOI:** 10.1038/s41598-020-78477-x

**Published:** 2020-12-09

**Authors:** Manal Matoug-Elwerfelli, Hani Nazzal, El Mostafa Raif, Stacy-Paul Wilshaw, Filomena Esteves, Monty Duggal

**Affiliations:** 1 Division of Oral Biology, School of Dentistry, University of Leeds, Leeds, UK; 2 Department of Clinical Dental Science, Princess Nourah Bint Abdulrahman University, Riyadh, Kingdom of Saudi Arabia; 3 Department of Paediatric Dentistry, School of Dentistry, University of Leeds, Leeds, UK; 4 Hamad Dental Centre, Hamad Medical Corporation, Doha, Qatar; 5 Institute of Medical and Biological Engineering, University of Leeds, Leeds, UK; 6 School of Pharmacy and Medical Sciences, University of Bradford, Bradford, UK; 7 Leeds Institute of Cancer and Pathology, St James University Hospital, Leeds, UK; 8 Discipline of Orthodontics and Paediatric Dentistry, Faculty of Dentistry, National University Singapore, Singapore, Singapore

**Keywords:** Biological techniques, Stem cells, Health care, Materials science

## Abstract

Implementing the principles of tissue engineering within the clinical management of non-vital immature permanent teeth is of clinical interest. However, the ideal scaffold remains elusive. The aim of this work was to assess the feasibility of decellularising rat dental pulp tissue and evaluate the ability of such scaffold to support stem cell repopulation. Rat dental pulps were retrieved and divided into control and decellularised groups. The decellularisation protocol incorporated a low detergent concentration and hypotonic buffers. After decellularisation, the scaffolds were characterised histologically, immunohistochemistry and the residual DNA content quantified. Surface topography was also viewed under scanning electron microscopy. Biocompatibility was evaluated using cytotoxicity assays utilising L-929 cell line. Decellularised scaffolds were recellularised with human dental pulp stem cells up to 14 days in vitro. Cellular viability was assessed using LIVE/DEAD stain kit and the recellularised scaffolds were further assessed histologically and immunolabelled using makers for odontoblastic differentiation, cytoskeleton components and growth factors. Analysis of the decellularised scaffolds revealed an acellular matrix with histological preservation of structural components. Decellularised scaffolds were biocompatible and able to support stem cell survival following recellularisation. Immunolabelling of the recellularised scaffolds demonstrated positive cellular expression against the tested markers in culture. This study has demonstrated the feasibility of developing a biocompatible decellularised dental pulp scaffold, which is able to support dental pulp stem cell repopulation. Clinically, decellularised pulp tissue could possibly be a suitable scaffold for use within regenerative (reparative) endodontic techniques.

## Introduction

Preserving pulp vitality, in pulpally compromised teeth, is the ultimate goal in dentistry^1,2^, however; despite our best efforts such goal is not always achievable. The management of teeth with necrotic pulps is extremely challenging especially in immature teeth with compromised crown root ratio, and thin dentinal walls. The use of a bioengineered treatment strategy allowing pulpal regeneration and continuation of root development, therefore, is highly advantageous^3,4^. Consequently, careful consideration and incorporation of bioengineering principles (the use of stem cells, scaffold material and morphogenic signals) within such a treatment strategy is essential in the clinical application of regenerative^5–7^, or more accurately termed reparative^8^, endodontics.

Several natural and synthetic scaffold materials have been proposed for regeneration of the pulp dentine complex, however, the ideal scaffold is yet to be identified^5,9^. A recent review of the literature highlighted a large heterogeneity of results on several tested scaffold materials, highlighting the need to develop a new scaffold material with close resemblance to the natural pulp matrix^10^.

More recently, the use of biological scaffolds derived through tissue decellularisation has been advocated^11,12^. Ideally, the produced decellularised scaffold should be composed of an acellular extracellular matrix (ECM) with a preserved three-dimensional histological architecture and natural structural components^13^. The ECM is a vital and dynamic structure composed of tissue specific structural and functional proteins. This complex integration between cells and the surrounding microenvironment (ECM proteins) regulates cellular activity and may provide a suitable environment for tissue regeneration^14^.

Biological scaffolds derived from tissue decellularisation have been assessed for dental applications including decellularisation of bovine pulp tissue^15^, porcine tooth buds^16,17^, swine pulp tissue^18,19^, human root slices^20^, and human pulp tissue^6^. While successful biocompatible decellularisation have been shown in human pulp tissues^6^, further research is required to show the decellularised scaffolds’ ability to support stem cell recellularisation and differentiation into odontoblast-like cells.

Decellularised rat tissues, as experimental models, have been reported in the literature^21,22^, and can be regarded as an alternative research tissue source to human tissues which are becoming increasingly difficult to obtain. Of interest, histological characterisation of rat dental pulp tissues has shown similar structural composition to human pulp tissues^23,24^. Therefore the aim of the present work was to illustrate the ability to decellularise rat pulp tissues using low concentration detergents and evaluate the ability of such decellularised scaffold to support stem cell growth and differentiation.

## Materials and methods

### Ethical consideration

All experimental protocols and study design was approved by the Research Ethics Committee, University of Leeds, United Kingdom (approval no. 101013/MME/113). The use of human and animal tissue in this study followed relevant guidelines and regulations as set by University of Leeds Research Ethics Committee.

### Tissue procurement

Rat jaws were only collected for this study. Wistar male rats (28 ± 2 days old) were humane killed in-advance in the Central Biomedical Services (University of Leeds, Leeds, United Kingdom) as per the institutional animal use and care committee at the University of Leeds and according to guidelines laid down by the Animals (Scientific Procedures) Act 1986. Dental pulp tissues were aseptically retrieved from the rat mandibular incisors and stored in 0.1 M phosphate-buffered saline (PBS, 17-516F, Lonza, Slough, United Kingdom; pH 7.4) at − 80 °C for up to 4 weeks. All reagents and solutions were purchased from Sigma-Aldrich (Poole, United Kingdom) and cell culture plastics were obtained from Corning, Inc. (Amsterdam, The Netherlands) unless stated otherwise.

### Decellularisation of dental pulp

Tissue decellularisation was performed as described by Matoug-Elwerfelli et al. (2018). Under sterile conditions, pulp tissues were thawed and processed individually in 4 mL solutions and the following protocol was performed. Initially, pulp tissues were incubated in hypotonic Tris-buffer (10 mM Tris, T3038, pH 8.0) containing protease inhibitors [(0.1%, w/v ethylenediaminetetraacetic acid; EDTA, 036980 and (10 KIU/mL aprotinin, A6279)] overnight at 4 °C. Pulp tissues were then subjected to a single cycle of a hypotonic buffer containing 0.03% (w/v) sodium dodecyl sulphate (SDS, 71736) and protease inhibitors for 24 h at room temperature with agitation. Pulp tissues were washed three times in Tris-buffered saline (pH 7.4, T5912) with agitation. Following washing, pulp tissues were incubated in 50 U/mL DNase (AMPD1) and 1 U/mL RNase (R4642) in a buffer [(50 mM Tris-hydrochloric acid (93313), 10 mM magnesium chloride (M1028), and 50 µg/mL bovine serum albumin (A7979) pH 7.5] for 3 h at 37 °C with agitation. Pulp tissues were subsequently washed in Tris-buffered saline (pH 7.4) with agitation. Incubation in 0.1% (v/v) peracetic acid (77240) in PBS for 3 h served as a final disinfection. Finally, tissues were washed three times in Tris-buffered saline (pH 7.4) for 1.5 h at room temperature with agitation.

### Tissue preparation

Samples of control and decellularised rat pulps (*n* = 4 per group) were fixed in 10% (w/v) neutral buffered formalin (BAF-6000-08A, CellPath Ltd, Newtown, United Kingdom) for 24 h. Fixed tissues were embedded in 2% (w/v) agar (A-9915), dehydrated in an automated tissue processor (ASP200, Leica Biosystems Newcastle Ltd, Newcastle Upon Tyne, United Kingdom), embedded in paraffin wax and serially sectioned at 5 μm. These sections were then used for the following histology and immunohistochemistry analysis.

### Histology analysis

Paraffin embedded tissue sections were dewaxed utilising 4 changes of xylene (X/0250, Thermo Fisher Scientific, Loughborough, United Kingdom) for 5 min each and rehydrated using descending ethanol series [100, 90 and 75% (v/v) for 2 min each]. Following dewaxing and rehydration, tissue sections of control and decellularised pulp tissues were stained using standard histological procedures with haematoxylin and eosin (H&E; RRSP60-D, Thermo Fisher Scientific), 4′,6-diamidino-2-phenylindole (DAPI, H-1200, Vector Laboratories Ltd, Peterborough, United Kingdom), alcian blue (RRSK400, TCS Biosciences Ltd, Buckingham, United Kingdom), and picrosirius red (24901, Polysciences, Inc. Northampton, United Kingdom). Following staining; the slides were dehydrated using ascending ethanol series [75% for 5 min and 3 changes of 100% (v/v) for 20 s], cleared in four successive changes of xylene for 5 min each and then mounted with synthetic resin media (DPX mountant, REA212, Solmedia Ltd, Shrewsbury, United Kingdom). All slides were viewed using either normal Köhler illumination, polarised light or under reflected light and a DAPI filter (*λ*_*ex*_ = 365 nm/*λ*_*em*_ = 445/450 nm) with images captured digitally using an AxioCam and AxioVision or Zen software (ZEISS, Cambridge, United Kingdom) and qualitatively assessed.

### Immunohistochemistry analysis

Following dewaxing and rehydration (as mentioned above), antigen retrieval methods were performed using either; (1) Bond enzyme pre-treatment kit (AR9551, Leica Biosystems Newcastle Ltd.) by incubating slides for 15 min at 37 °C or (2) Heat-induced antigen unmasking solution [Tris–EDTA buffer solution (pH 9.0) or citric acid buffer solution (pH 6.0)] (Vector Laboratories Ltd.) in an automated pressure cook for 2 min under full pressure as per manufacturer’s instructions. Following antigen retrieval, slides were then incubated at room temperature for 1 h with the following primary antibodies; mouse monoclonal antibodies against collagen type I (1:100, ab90395, Abcam, Cambridge, United Kingdom), collagen type III (1:200, ab6310, Abcam), fibronectin (1:50, ab6328, Abcam,), Alpha-1,3 Galactose (α-gal, 1:200, ALX-801–090, Enzo Life Sciences, Inc., Lausen, Switzerland), major histocompatibility class II RT1B (1:200, Bio-Rad Laboratories Ltd, Hertfordshire, United Kingdom), and rabbit polyclonal laminin antibody (1:400, NB300-144, Novus Biologicals, Abingdon, United Kingdom) were used to assess collagen matrix, xenoepitope expression, specific cellular and basement proteins components. Secondary antibody staining (immunolabelling) using either ImmPRESS Excel peroxidase anti-mouse (MP-7602, Vector Laboratories Ltd.) or horseradish peroxidase anti-rabbit polymer (MP-7401, Vector Laboratories Ltd.) were used following manufacturer’s instructions. Tris-buffered saline (pH 7.4) was used throughout as the diluent and wash buffer. Non-specific background staining was prevented by endogenous hydrogen peroxide blocking with Bloxall (SP-6000, Vector Laboratories Ltd.) and protein blocking (1/10 casein, Ssp-5020, Vector Laboratories Ltd.). Next, the slides were washed twice with Tris-buffered saline with 0.1% (v/v) Tween 20 (pH 7.6; T9039) and stained with ImmPACT 3,3′-diaminobenzidine (DAB) peroxidase chromogen substrate (SK-4105, Vector Laboratories Ltd.) for 5 min. Omission of the primary antibody served as the negative control. Isotype control antibodies [(IgG, IgG1; Abcam), (IgG1 Kappa, IgG2b Kappa; Sigma-Aldrich), (IgM; Novus Biologicals), (IgG2a Kappa; Dako, Agilent Technologies Ltd. Cheshire, United Kingdom)] were used to verify antibody specificity. Following antibody labeling all sections were counter stained using Mayer’s hematoxylin, dehydrated, cleared and mounted (as mentioned above). All slides were viewed under Köhler illumination with images captured digitally using an AxioCam and AxioVision software (ZEISS) and qualitatively assessed.

### DNA quantification assay

Total DNA was extracted using a DNA isolation kit for tissues (69504, QIAGEN Ltd. Manchester, United Kingdom). Briefly, control and decellularised dental pulp tissues (*n* = 4 per group) were digested using a Proteinase K solution. Samples were loaded onto spin columns and washed to remove protein and other contaminates. The DNA was eluted into DNase- and RNase-Free Eppendorf tubes and quantified using a NanoDrop Spectrophotometer at 260 nm (ND-2000C, Thermo Fisher Scientific).

### Scanning electron microscopy

Control and decellularised pulp tissues (*n* = 4 per group) were fixed in 2.5% (v/v) glutaraldehyde, washed in distilled water and dehydrated using ascending ethanol concentrations. Specimens were treated with 50% and 100% (v/v) hexamethyldisilazane solution and left to evaporate overnight. Specimens were then mounted on aluminium stubs, coated with gold in sputter coating unit and visualised under conventional high vacuum scanning electron microscope (SEM; Hitachi S-3400 N, High-Technologies, Ltd.) to evaluate tissue surface topography. All images were captured digitally.

### Cell culture

L-929 murine fibroblasts (Health Protection Agency) were cultured in Dulbecco’s modified Eagle’s medium (DMEM, D6546) containing 10% (v/v) foetal calf serum (DE14-802), 100 U/mL penicillin, 100 µg/mL streptomycin (P4333), and 2 mM L-glutamine (G7513, Lonza) at 37 °C in 5% (v/v) CO_2_ in air; the medium was exchanged twice a week until the cells reached 80% confluency.

Human dental pulp stem cells (DPSCs) were obtained from Skeletal Research Tissue Bank, School of Dentistry, University of Leeds, following ethical approval (as mentioned above). DPSCs (passage 5) were cultured in alpha-modified Eagle’s medium (BE12-169F, Lonza) containing 10% (v/v) foetal calf serum, 100 U/mL penicillin, 100 mg/mL streptomycin, and 2 mM L-glutamine at 37 °C in 5% (v/v) CO_2_ in air; the medium was exchanged twice a week until the cells reached 80% confluency.

### Contact cytotoxicity assay

Contact cytotoxicity assay was performed in a similar manner to previous studies^13,25^. Samples of decellularised dental pulps (*n* = 4 per group) were attached to the centre of 6-well tissue culture plates using collagen type I (A10483-01, Gibco, Thermo Fisher Scientific). Cyanoacrylate glue (Z105902-1EA) or collagen type I in triplicate wells served as positive and negative controls, respectively. L-929 cells were seeded into each well at a density that would allow confluency after 48 h in cell standard culture medium. Plates were incubated at 37 °C in 5% (v/v) CO_2_ in air for 48 h. Following incubation, the culture medium was carefully aspirated before the wells were washed with PBS (pH 7.4) containing calcium and magnesium and fixed with 10% (v/v) neutral buffered formalin for 10 min. The wells were then stained with Giemsa solution (Merck R66 formulation, 352603R, VWR International, Ltd. Lutterworth, United Kingdom) for 5 min. The wells were rinsed with distilled water until clear and air dried. Changes in cell morphology and confluency were analysed using an inverted microscope under bright field illumination (Cell^B software) with all images captured digitally.

### Extract cytotoxicity assay

Segments of decellularised dental pulps (*n* = 8) were finely minced and left to incubate in DMEM (1 mL/mg) for 72 h at 37 °C with agitation. Following incubation, the mixture was centrifuged at 500* g* for 15 min and the supernatant was collected. L-929 cells were seeded onto 96-well plates (Optiplate white plates; PerkinElmer Ltd. Beaconsfield, United Kingdom) at a density that would allow confluency after 24 h in standard culture medium. Plates were incubated at 37 °C in 5% (v/v) CO_2_ in air. Following incubation, the culture medium was removed and replenished with 100 µL of fresh culture medium. For the study wells, 100 µL of decellularised extracts was added and further incubated for 24 h. 40% (v/v) dimethyl sulfoxide (DMSO, D8418) or DMEM in triplicate wells served as positive and negative controls, respectively. The cell viability (relative cellular Adenosine TriPhosphate [ATP] content) was then determined using the ATPlite Luminescence assay (6016941, PerkinElmer Ltd.) following manufacturer’s instructions.

### Recellularisation of the decellularised pulp tissue

The decellularised rat dental pulp tissues were recellularised with human DPSCs. Decellularised tissues (*n* = 8) were individually placed in a sterile 200 µL capacity Eppendorf tube supplemented with 180 µL of a diluted cell suspension containing 1 × 10^4^ cells. The Eppendorf tubes were sealed with OPSITE FLEXIGRID membrane (4628, Smith & Nephew, Watford, United Kingdom), placed within an in-house rotator apparatus (10 rpm) and incubated at 37 °C in 5% (v/v) CO_2_ in air for 24 h. The scaffolds were transferred into a 12-well culture plates, topped up with 2 mL of fresh cell culture medium. The culture medium was changed twice a week throughout the duration of the experiment (7 and 14 days).

### Determination of viability of human DPSCs cultured on decellularised pulp tissue

Following recellularisation, the ability of the decellularised scaffolds to support cell viability was analysed, at each time point, using a commercial LIVE/DEAD staining kit following the manufacturer’s instructions (Invitrogen L3224, Thermo Fisher Scientific). Stained scaffolds were then viewed under confocal Leica microscope (SP2 PLUS; Leica Microsystems, Milton Keynes, UK).

### Histological and immunohistochemistry assessment of human DPSCs cultured on decellularised pulp tissue

Following recellularisation, the ability of the decellularised scaffolds to support stem cell attachment, growth and differentiation were analysed using H&E and immunohistochemical staining methods (*n* = 4, each time point). Standard histology and immunohistochemical methods were performed as previously described above. Primary antibodies against odontoblastic markers included mouse monoclonal dentine matrix protein-1 (DMP-1, 73633, 1:100, Santa Cruz Biotechnology, Inc., Heidelberg, Germany), dentine sialophospoprotein (DSPP, 73632, 1:1000, Santa Cruz Biotechnology, Inc.), and rabbit polyclonal alkaline phosphatase (ALP, GTX100817, 1:1000, BD BioScience, Oxford, United Kingdom). Structural components included mouse monoclonal alpha-smooth muscle actin (α-SMA, ab7817, 1:500, Abcam), nestin (MAB1259, 1:5000, R&D Systems, Abingdon, United Kingdom), and vimentin (M0725, 1:10000, Dako). Growth factors including rabbit polyclonal vascular endothelial growth factor (VEGF-A, ab46154, 1:200, Abcam), and rabbit monoclonal vascular endothelial growth factor receptor-2 (VEGFR-2, 2479, 1:25, Cell Signaling Technology, Inc. Leiden, The Netherlands) antibodies were also assessed.

### Statistical analysis

Quantitative data was analysed using either an independent Student’s *t*-tests or one-way analysis of variance (ANOVA) depending on the number of groups. All experiments were performed in triplicates. The statistical analysis was performed using Prism 6 (GraphPad Software, California, USA), *p-*values < 0.05 were considered significant.

## Results

### Decellularisation assessment

**Figure 1:**
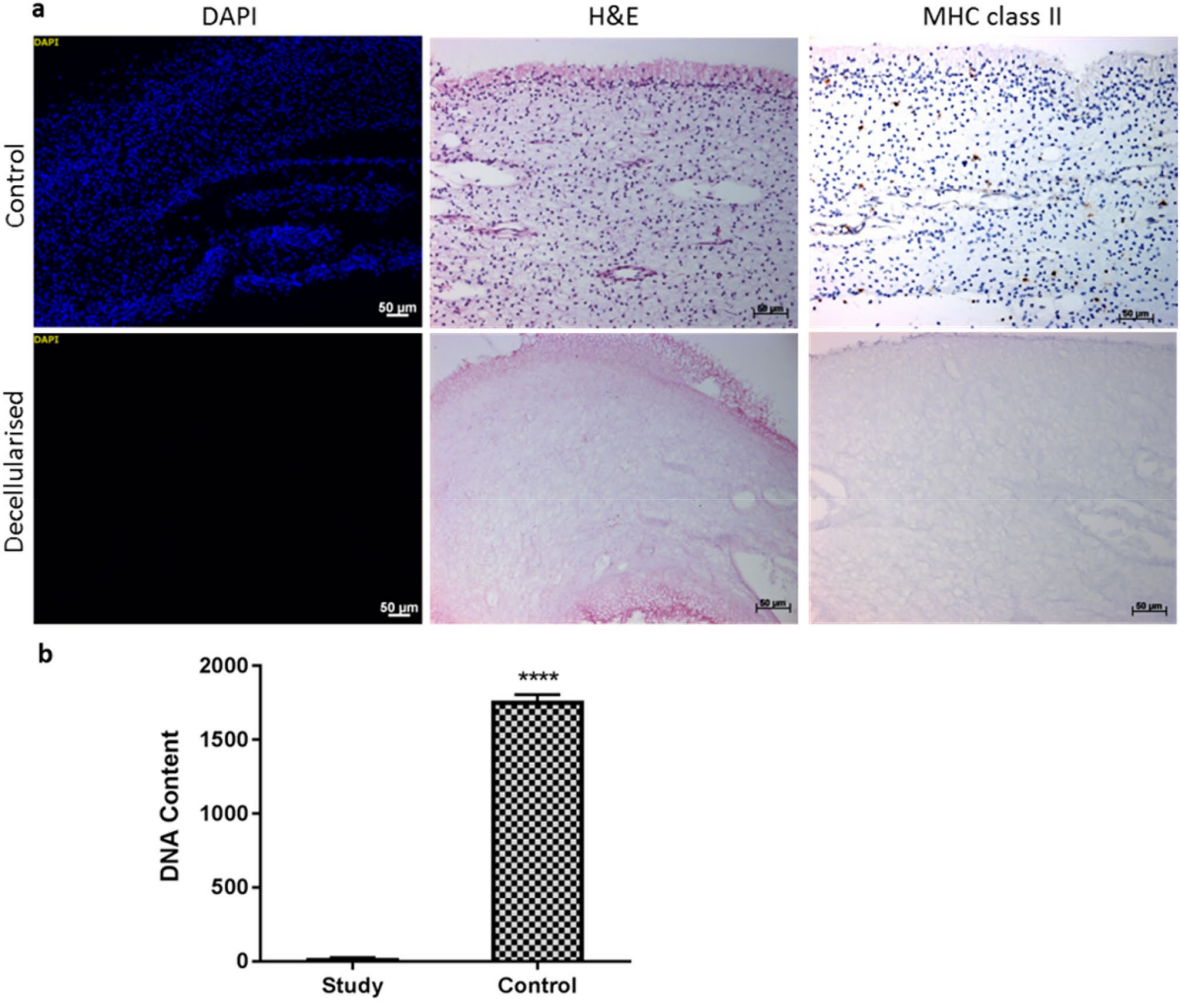
Decellularisation assessment. (**a**) Representative images of histological and immuolabelled rat pulp tissues. H&E and DAPI staining methods show highly cellular control tissues (nuclei stained blue) in comparison to no nuclear material staining of decellularised tissues. A positive immunoreactivity against major histocompatibility complex class II antigens were seen in control tissues, in contrast a negative immunoreactivity in decellularised tissues. (**b**) Bar graph results of DNA quantification assay determined by Nanodrop spectrometer. Y-axis; DNA content ng per mg of dry tissue. Data represent mean values (*n* = 4) ± 95% confidence intervals. The mean DNA measurements in the decellularised tissues contained 23.00 ± 1.43 ng/mg in comparison to 1756 ± 20.66 ng/mg in the control tissues (*t* test, ****p < 0.0001). *DAPI* 4′,6-diamidino-2-phenylindole. Scale bars are at 50 µm.

Decellularisation efficiency of rat dental pulp tissues was performed using both qualitative and quantitative assessment methods. Histology analysis using H&E and DAPI staining revealed a high density of cells within control (native) dental pulps and absence of cells in decellularised samples (Fig. 1a). Immunolabelling with major histocompatibility complex class II antibodies also revealed a positive immunoreactivity in the control pulp tissues, this positive staining was associated with specific cells scattered throughout the ECM (Fig. 1a). In contrast, a negative immunoreactivity was detected in the decellularised samples (Fig. 1a). No brown staining was seen in isotype stained sections (images not provided).

Quantitative analysis using total DNA from both control and decellularised pulp tissues was extracted and quantified. The mean DNA content in the decellularised tissues was 23.00 ± 1.430 ng DNA per mg of dry tissue, in comparison to the 1765 ± 20.66 ng DNA per mg of dry control tissues. This reduction of over 98% was found to be significantly different when compared to the control tissue (Fig. 1b).

### Collagen structure assessment

**Figure 2:**
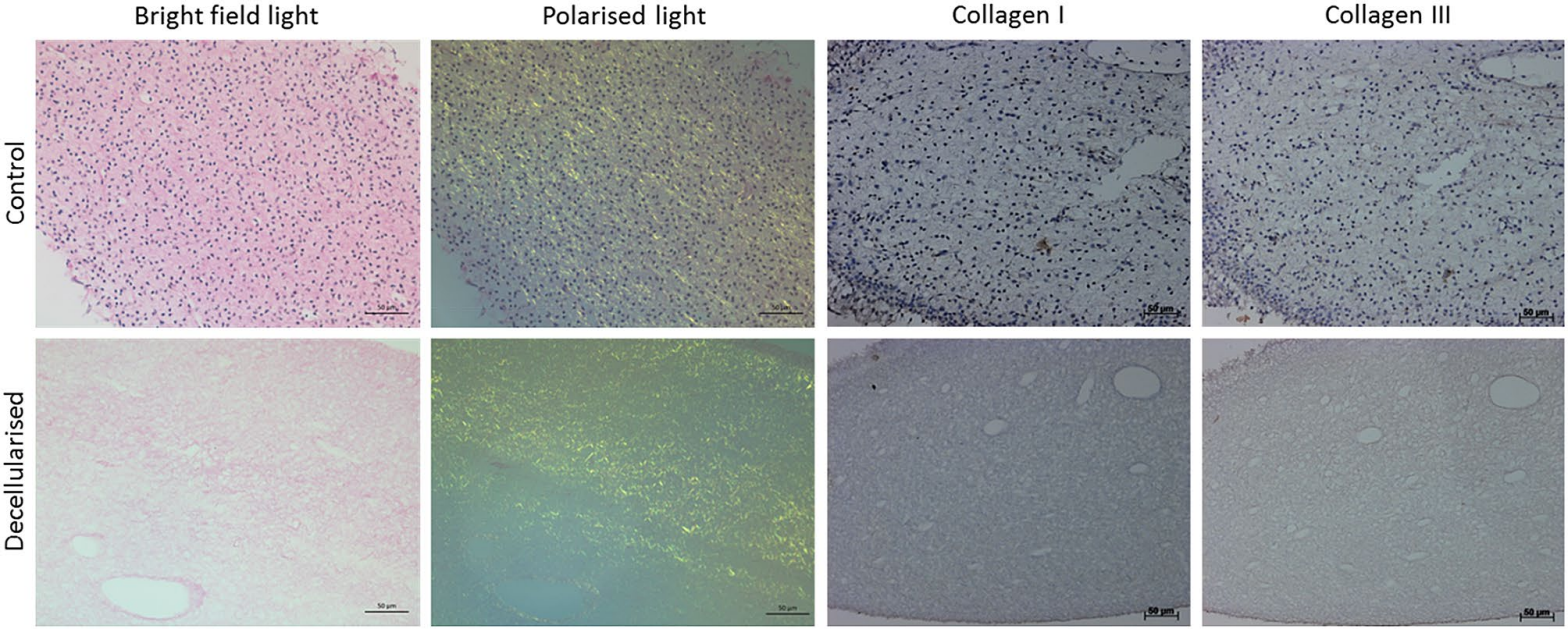
Collagen structure assessment. Representative images of rat dental pulp tissues stained with picrosirius red, labelled with collagen type I and III antibodies. Control tissues reveal a porous collagen matrix, mainly composed of immature collagen fibres (stained green under the polarised light), and a weak positive immunoreactivity to collagen type I and III antigens. Following decellularisation, preservation of an acellular porous collagen matrix and a positive, but reduced, staining pattern of collagen type I and III fibres in comparison to the control tissues was evident. Scale bars are at 100 and 50 µm.

Assessment of the collagen structure of rat dental pulp tissues was performed using both histology and immunohistochemistry analysis. Histological characterisation of picrosirius red stained control tissues viewed under both bright field and polarised light microscopy revealed a collagen matrix mainly composed of thin filaments and fibrils that were weakly birefringent to light (Fig. 2). Following decellularisation, the collagen fibre network was overall well preserved throughout the entire matrix (Fig. 2). Antibody labelling against collagen type I and type III was additionally performed (Fig. 2). In control tissues, a weak positive immunoreactivity to collagen type I and III antigens was observed. These thin fibres and fibrils were distributed throughout the matrix, mainly concentrated around and along the vasculature. Following decellularisation, a positive but less intense staining pattern was seen (Fig. 2). No brown staining was seen in isotype stained sections (images not provided).

### Non-collagenous structures assessment

**Figure 3:**
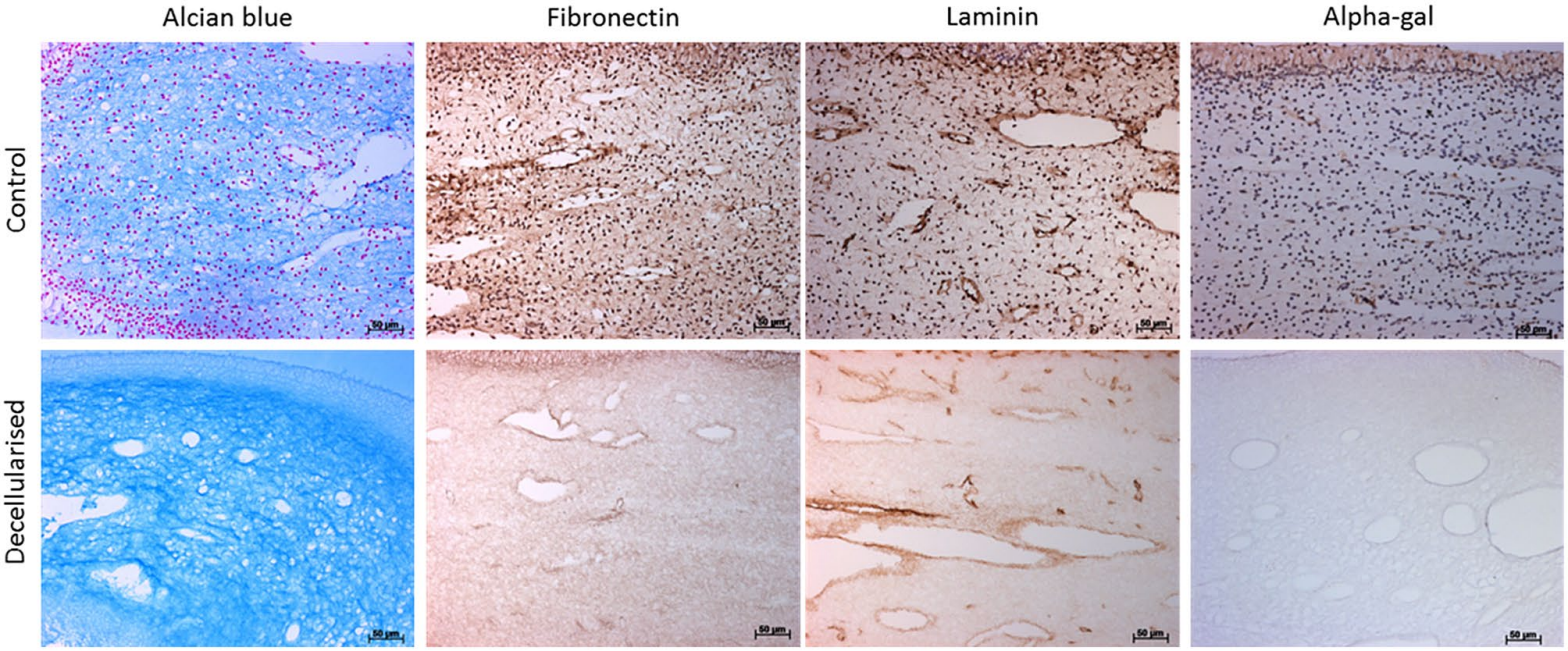
Non-collagenous structures assessment. Representative images of rat dental pulp tissues stained with alcian blue, labelled with fibronectin, laminin and α-gal antibodies. Control tissues reveal a rich glycosaminoglycans rich matrix and a positive immunoreactivity to fibronectin, laminin proteins and α-gal. Following decellularisation, preservation of glycosaminoglycans, fibronectin and laminin proteins with negative α-gal staining was evident. *α-gal* alpha-1,3 galactose. Scale bars are at 50 µm.

Assessment of the non-collagenous ECM components of rat dental pulp tissues was performed using histology and immunohistochemistry analysis. Histological characterisation of alcian blue stained control tissues revealed a glycosaminoglycan rich tissue (Fig. 3). Following decellularisation, preservation of glycosaminoglycan molecules was evident with no visible differences between the control and the decellularised tissues (Fig. 3). Antibody labelling against structural proteins and α-gal was performed (Fig. 3). In control tissues, fibronectin and laminin appeared as a fibrous plexus mainly concentrated around the vasculature. Following decellularisation, a positive but noticeably weaker staining pattern against fibronectin was observed. Although laminin staining was less intense in comparison to the control tissues, the distribution was comparable to control tissues, it was found to be present throughout the entire ECM mainly concentrated around the basement membrane of vasculature structures. No evidence of α-gal was observed within the decellularised ECM (Fig. 3). No brown staining was seen in isotype stained sections (images not provided).

### Scanning electron microscopy

**Figure 4:**
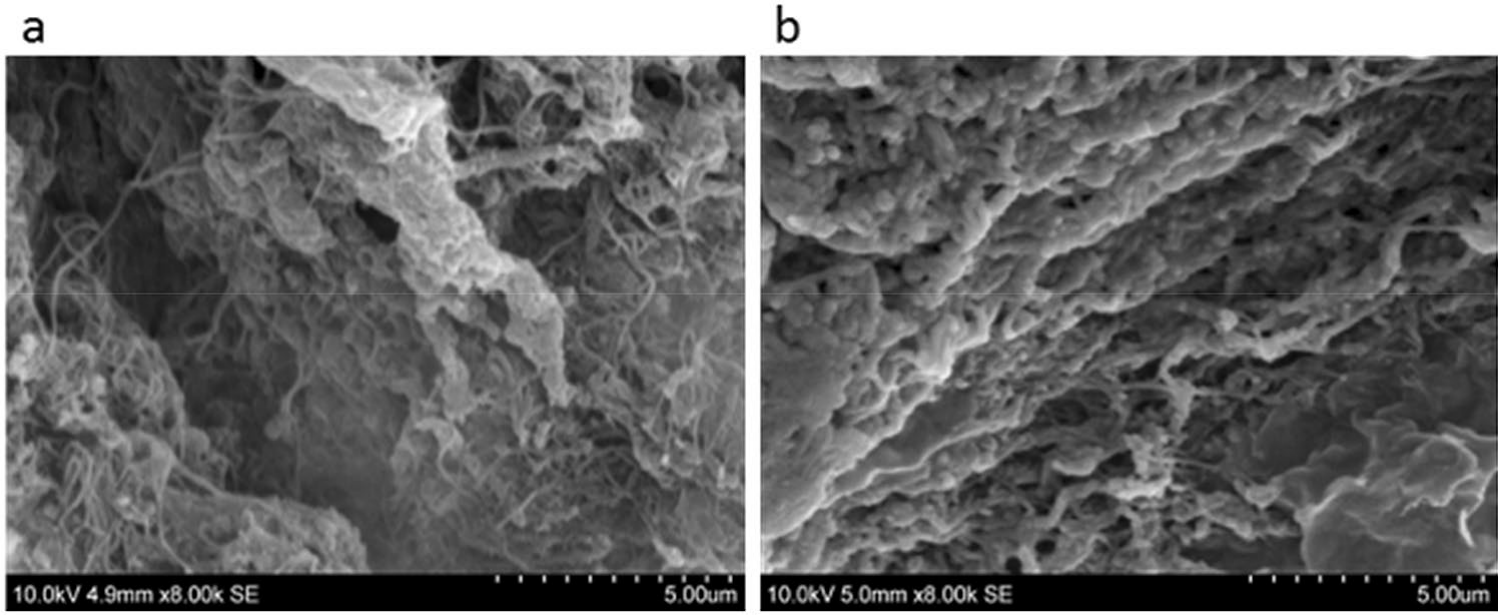
Scanning electron microscopy surface assessment. (**a**) Control rat dental pulp tissues reveal a complex irregular fibre mesh structure. (**b**) Decellularised tissue show structural preservation. Scale bar at 5 µm.

Structural assessment of rat dental pulp analysed under SEM revealed a complex irregular fibre mesh structure related to the control tissues (Fig. 4a). Following decellularisation, preservation of complex fibre mesh denuded of some components, mainly cell and cell fragments was evident (Fig. 4b).

### Contact cytotoxicity assay

**Figure 5:**
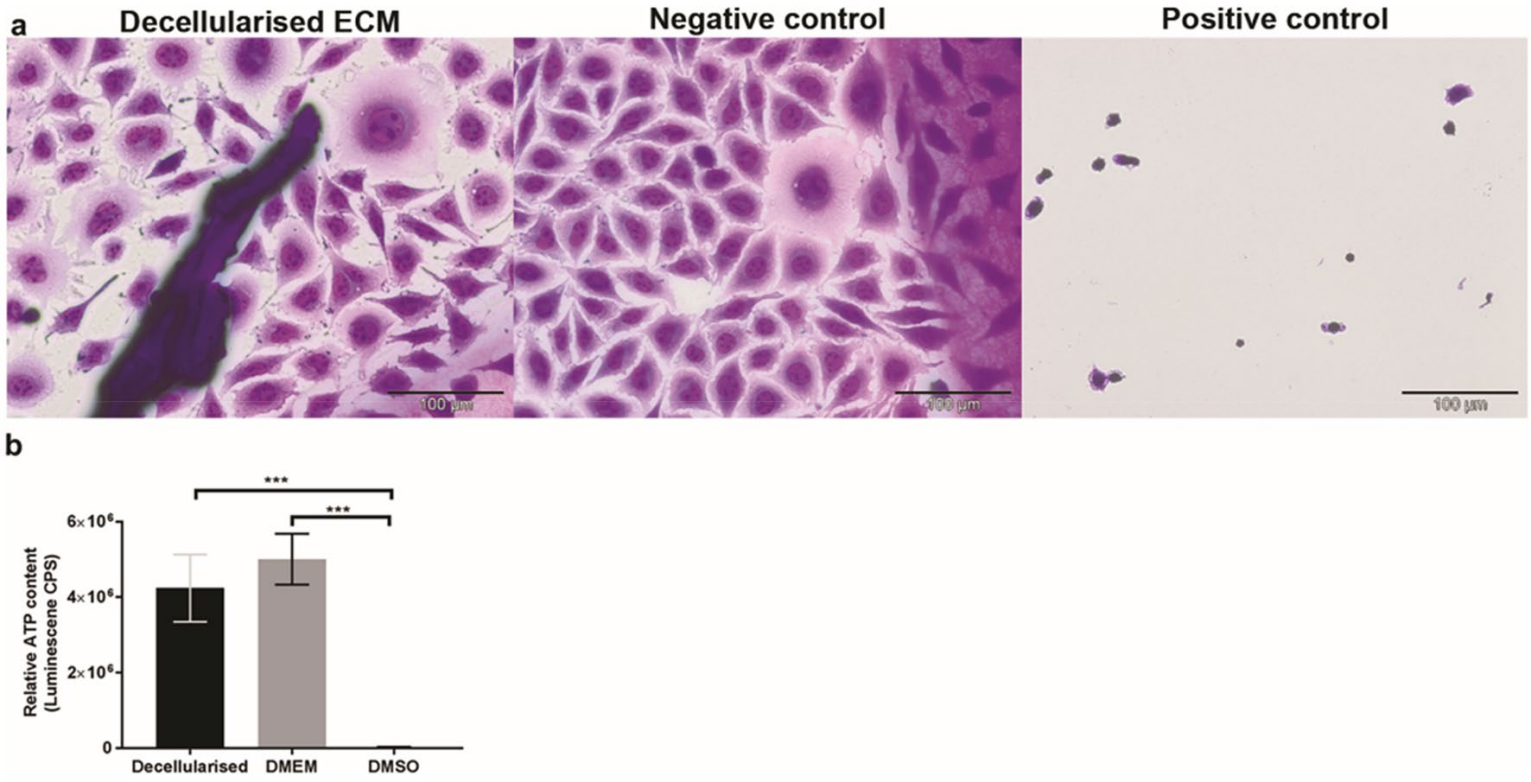
Cytotoxicity analysis of decellularised rat pulp tissue. (**a**) Microscopic analysis of contact cytotoxicity assay cultured with L-929 cells and stained with Giemsa stain. Decellularised tissues resulted in no obvious cytotoxicity with cell growth in contact with the pulp tissue. Collagen gel (negative control) resulted in no cytotoxicity. Cyanoacrylate glue (positive control) resulted in marked cytotoxicity. (**b**) Bar graph results of extract cytotoxicity assay measuring the relative cellular ATP content of L-929 cells. Y-axis relative cellular ATP measurements. Data represent mean values (*n* = 8) ± 95% confidence intervals. Data analysis revealed no significant difference between decellularised extracts and DMEM (negative control) and statistical significance in comparison with DMSO (positive control) (ANOVA, ****p < 0.0001). *ATP* adenosine-5-triphosphate, *DMEM* Dulbecco’s modified Eagle’s medium, *DMSO* dimethyl sulfoxide, *ECM* extracellular matrix. Scale bars are at 100 µm.

Microscopic analysis of the cell culture plates showed L-929 cell growth up to and in contact with the samples of decellularised dental pulp (Fig. 5a). No obvious morphological change nor cell lysis were noted. Collagen gel alone (negative control) showed no signs of cytotoxicity, while cyanoacrylate glue (positive control) resulted in cell lysis.

### Extract cytotoxicity assay

There was no significant reduction in the cellular ATP levels of the L-929 cells following incubation with soluble extracts of decellularised dental pulps compared with that of the DMEM (negative control) (ANOVA, *p* > 0.05; Fig. 5b). In contrast, DMSO (positive control) caused an almost total loss of ATP and were significantly lower than any other sample group (ANOVA, *p* < 0.0001; Fig. 5b).

### Determination of viability of human DPSCs cultured on decellularised pulp tissue

**Figure 6:**
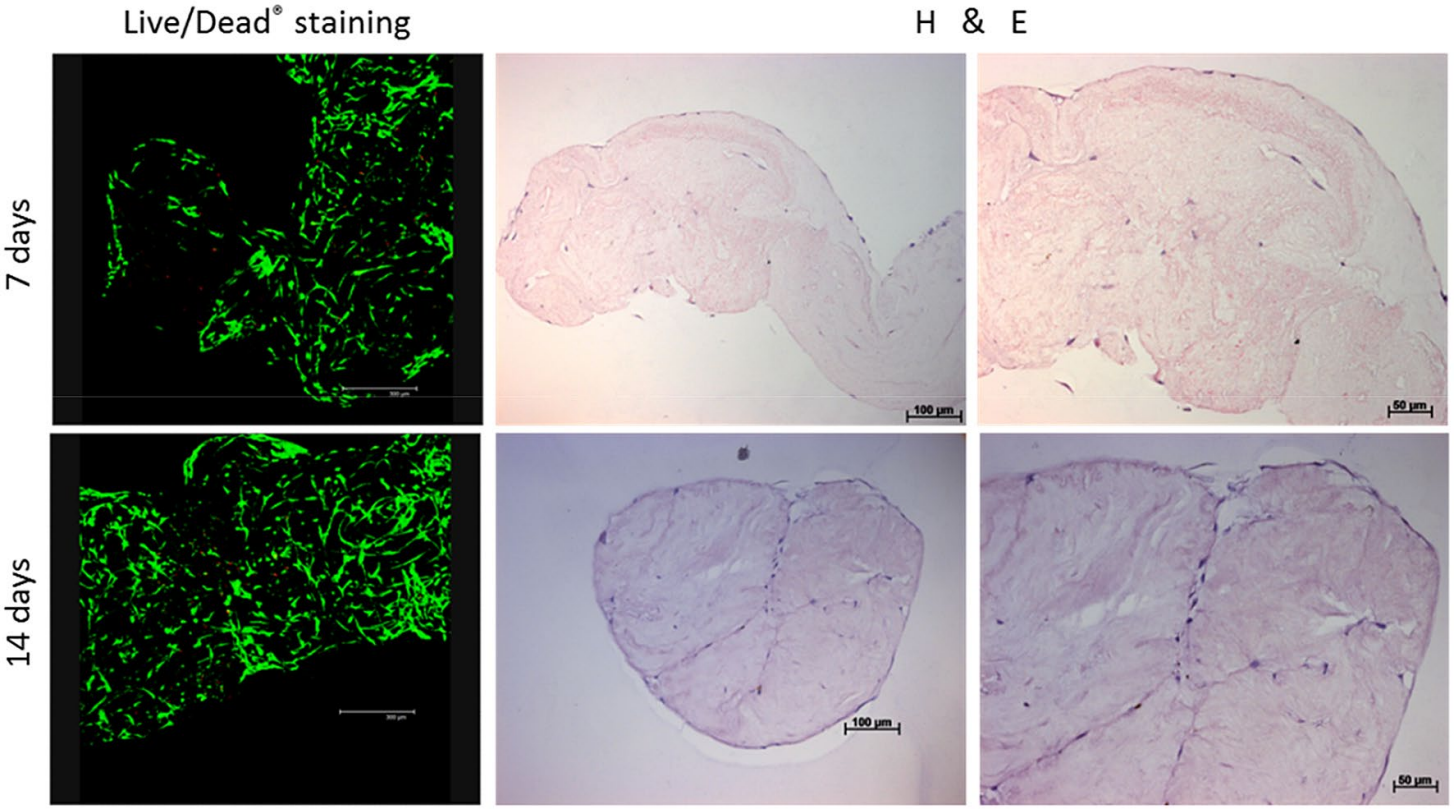
DPSCs viability and attachment following recellularisation of the decellularised pulp scaffold. (**a**) Confocal laser microscope images of cell viability assay following 7 and 14 day culture clearly show a well-populated scaffold with viable cells **(**stained green) and scarce dead cells (stained red). Scale bars are at 300 µm. (**b**) Representative images of H&E staining revealed DPSCs attached and migrated within the decellularised scaffold. As culture time increased, the seeded scaffold contracted from longitudinal shape (day 7) to a circular shape (day 14). *DPSCs* dental pulp stem cells. Scale bars are at 100 and 50 µm.

The viability of DPSCs following recellularisation on the decellularised scaffolds was assessed using standard LIVE/DEAD staining kit. Following 7 and 14 days culture, Z-stack constructed confocal laser images clearly demonstrated viable cells (stained green with Calcein-AM), of elongated morphology, repopulating the decellularized scaffolds with limited appearance of dead cells (stained red with ethidium homodimer) (Fig. 6).

### Histological assessment of human DPSCs cultured on decellularised pulp tissue

Serial sections of the recellularised scaffolds were assessed with H&E staining to determine the ability of DPSCs to attach, migrate and differentiate within the decellularised scaffolds. Following 7 and 14 days culture, the DPSCs appeared elongated with a spindle-shaped morphology attached to the outer scaffold surface and between the collagen fibers. As time elapsed, there was an apparent change in the scaffold shape from a longitudinal (day 7) to a contracted circular shape (day 14; Fig. 6).

### Immunohistochemistry assessment of human DPSCs cultured on decellularised pulp tissue

**Figure 7:**
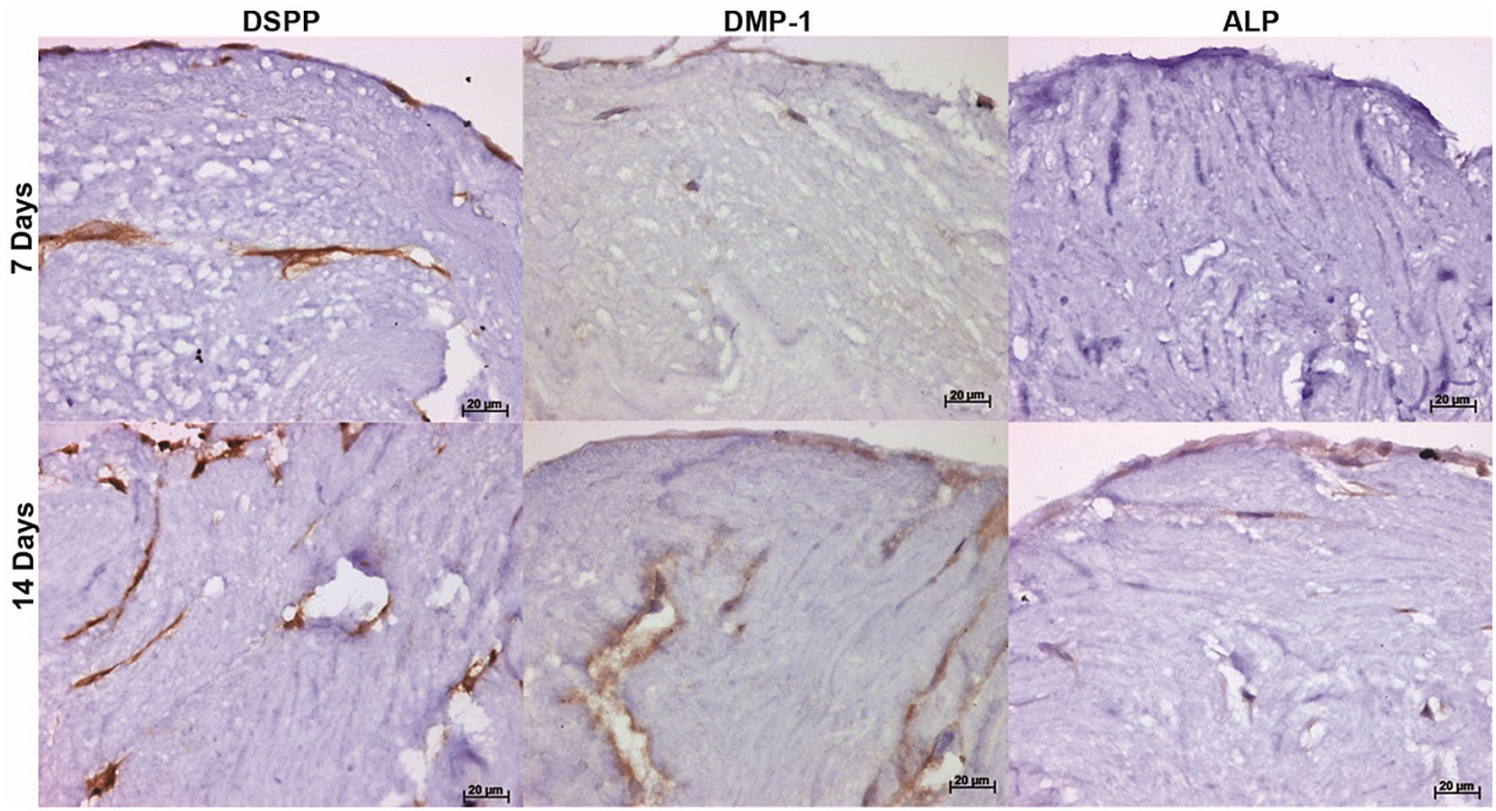
Odontoblastic markers following recellularisation of the decellularised pulp scaffold. Representative images of immunohistochemical labelled tissues showing a positive immunoreactivity against DSPP positive cells at 7 and 14 days culture. DMP-1 and ALP, were weakly stained at day 7 with an increase in staining intensity at day 14. *DSPP* dentine sialophospoprotein, *DMP-1* dentine matrix protein 1, *ALP* alkaline phosphatase. Scale bars are at 20 µm.

Antibody labelling was performed to assess the ability of the seeded DPSCs to express cytoskeleton components, growth factors and odontoblastic markers. Odontoblastic markers, DMP-1 and ALP, were weakly stained at day 7 with an increase in staining intensity following longer culture duration (day 14; Fig. 7). In contrast, DSPP was strongly positive following 7 and 14 days culture (Fig. 7).

**Figure 8:**
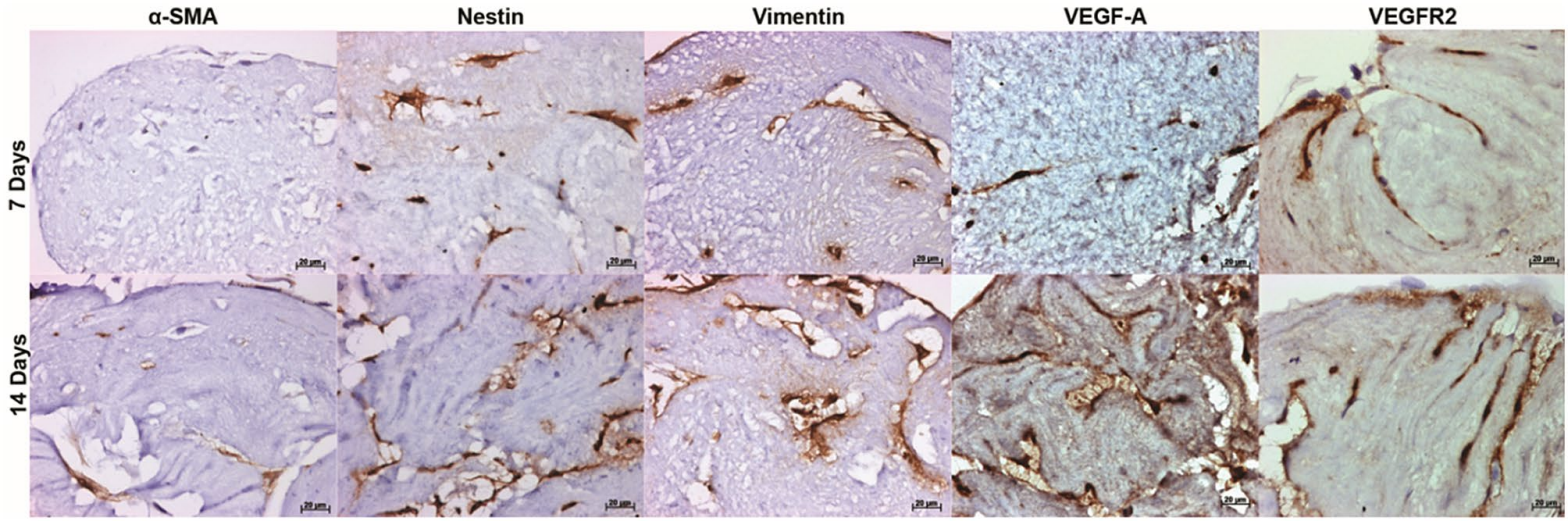
Gene expression following recellularisation of the decellularised pulp scaffold. Representative images of immunohistochemical labelled tissues revealed a positive immunoreactivity against nestin, vimentin, VEGF-A, and VEGFR-2 positive cells at 7 and 14 days culture. α-SMA was weakly stained at day 7 with an increase in staining intensity at day 14. *α-SMA* alpha smooth muscle actin, *VEGF-A* vascular endothelial growth factor-A, *VEGFR-2* vascular endothelial growth factor receptor 2. Scale bars are at 20 µm.

The seeded DPSCs were stained dark brown against vimentin, nestin, VEGF-A, and VEGFR-2 antigens indicating strong positive cellular immunoreactivity. These antigen positive cells were scattered throughout the scaffold at day 7 and 14 culture (Fig. 8). However, α-SMA was weakly stained at day 7 with an increase in staining intensity following 14 days culture (Fig. 8). No brown staining was seen in isotype stained sections (images not provided).

## Discussion

Scaffold development for use in regenerative (reparative) endodontics is currently the subject of intense research in an attempt to improve the clinical outcomes and prognosis of these vulnerable teeth. The ECM is regarded as a vital component of all tissues and, possibly, the ideal scaffold for tissue engineering^14^. Therefore, developing a biocompatible decellularised ECM scaffold is deemed likely to allow the ideal environment for stem cell repopulation and differentiation.

The decellularisation protocol performed in this work, involved cell membrane lysis in a hypotonic Tris buffer, solubilisation of cytoplasmic and nuclear components using a single detergent cycle of 0.03% SDS, followed by removal of cellular material with a nuclease treatment.

Rat dental pulp tissues were used in this work due to histological structural similarities to human pulp tissue^23,24^. The use of laboratory rats is also well known as inevitable part of translational biomedical research^26^, and considered an alternative tissue source due to the limited availability of human pulp tissues for experimental studies.

Collagen fibres are known as the most abundant fibrous component of the dental pulp^27^, and largely contribute towards the development and maintenance of the pulp matrix^28^. Analysis of the dried pulp tissue obtained from rat incisors revealed a low collagen content of around 3.5%^23^, with collagen type I and III representing the main bulk of collagen^23,29,30^. This low collagen content was in line in with our results showing immature collagen fibres stained green as viewed under the polarised light and a weak positive immunoreactivity to collagen type I and III antigens within the control tissues. Surface topography assessment of control rat dental pulp viewed under SEM revealed a complex irregular fibre mesh structure. Following decellularisation, preservation of structural collagen was evident with various qualitative methods utilized.

Therefore, in this work decellularised rat dental pulp tissues resulted in a biocompatible acellular scaffold with preservation of the connective tissue architecture, retained vascular channels and ECM proteins similar to those obtained following decellularisation of the human dental pulp tissue^6^.

Xenogenic hyperacute rejection is reported to be mediated by complement activation of antibodies against α-gal epitope^31,32^. Therefore, complete removal of α-gal epitope from the xenogenic tissue is essential. The results of this work showed no evidence of α-gal expression following immunohistochemical assessment of decellularised rat pulp tissues. These results are in line with those reported following decellularisation of porcine medial meniscus^33^, porcine bone cartilage^34^, and porcine pulmonary valve^25^, using comparable decellularisation protocols. A negative immune-reactivity against major histocompatibility complex class II molecules was also evident following decellularisation. These specific class of molecules are expressed by immune cells such as monocytes, macrophages and dendritic cells and serve as initial defence cells^35,36^.

Following recellularisation with DPSCs, the produced ECM scaffolds were able to support stem cell viability, attachment and migration in vitro. As time elapsed, a decrease in the overall size of the seeded scaffold was apparent, changing from original tubular shape observed at day 7 to a circular shape at day 14. Contraction of cell-seeded scaffolds has been reported in the literature following recellularisation of polyglycolic acid matrices with human fibroblastic cells^37^, and collagen-glycosaminoglycan matrices seeded with DPSCs^38^. This matrix contraction was thought to be linked to the expression of α-SMA positive cells^38^. The expression of α-SMA by DPSCs cultured on rat decellularised scaffolds, demonstrated in this study, is likely to have contributed to the contraction of the scaffolds. Further work is needed to assess this hypothesis in addition to the effects of such contraction on cell penetration, survival and differentiation.

In the present study, immunohistochemical labelling of recellularised rat scaffolds showed a positive expression against several genes and odontoblast markers. DPSCs seeded within the decellularised scaffolds expressed putative markers of odontoblastic differentiation including DSPP, DMP-1 and ALP following 14 days culture in standard medium conditions. These markers, although non-specific, have been recommended in identifying cell differentiation towards odontoblastic-like cell lineage^39,40^. Previous in vivo studies assessing the odontoblastic differentiation of stem cells reported a positive expression to DSPP and DMP-1 following 21 days culture when Puramatrix was seeded with either DPSCs in tooth slice model^41^, or stem cells from human exfoliated deciduous teeth in full length root canals^42^. Furthermore, human recombinant collagen scaffolds seeded with stem cells from human exfoliated deciduous resulted in a positive expression for DSPP and DMP-1 following 14 and 28 days culture, respectively^42^. Interestingly, odontoblastic differentiation of stem cells was not possible in both seeded scaffolds without the surrounding dentine structure^41,42^. Song et al*.* (2017) also reported positive expression of odontoblastic markers following 14 days cell culture utilizing a decellularised pulp tooth slice model^20^. In contrast, the present study revealed stem cell differentiation solely within the decellularised scaffold. Indeed, tooth specific environmental cues have a significant impact on the differentiation ability of stem cell into odontoblastic lineages^43–45^. Hence, it could be hypothesised, that placement of the decellularised scaffold in direct contact with the surrounding tooth structure enhances the tissue engineering process, with new tissue formation in close resemblance to the control tissue.

Successful engineering of a pulp-dentine complex also depends on angiogenesis and neurogenesis. Nestin, an intermediate filament protein, is classified as a neural progenitor marker and has been found to be expressed in newly differentiated and functioning odontoblast cells^46,47^. Vimentin, a major intermediate filament protein, has also been linked to neurogenesis and expressed by neuronal precursor cells^48^. Although vimentin is not considered as a pulp tissue specific protein, it has been suggested as a dental pulp quality standard protein for characterisation of pulp regeneration^49^. Indeed exposure of DPSCs to the correct environmental cues is crucial for their ultimate differentiation into active neurons^50^. Angiogenesis, on the other hand, aids in the transport of oxygen, nutrients and in attracting prevascular stem cells to the regenerated site, thereby preventing tissue necrosis^51,52^. Notably, several studies highlighted the ability of DPSCs in inducing angiogenesis and the expression of proangiogenic factors including VEGF-A and VEGFR-2^53,54^. The immunolabelling results of this study showed positive cellular expression against nestin, vimentin, VEGF-A, and VEGFR-2 antigens following recellularisation indicating, a possibility, of active neurogenesis and angiogenesis.

In conclusion, results of this work indicate successful decellularisation of rat dental pulp tissues utilising a single low concentration detergent based decellularisation protocol. The resulting biocompatible scaffold was able to support dental pulp stem cell repopulation over a 14 day period. The recellularised scaffold also showed a positive cellular expression against odontoblastic markers, cytoskeleton proteins, and growth factors.

Additional scaffold testing utilizing ex vivo culture models; tooth slice^55^ and ectopic root transplantation models^56,57^ in order to assess scaffolds ability to regenerate within the surrounding environment is of importance for future clinical translation^58^. Following animal testing, all work will be duplicated on human tissue for future clinical trials.
